# Dialysis outcomes in those aged ≥65 years

**DOI:** 10.1186/1471-2369-14-175

**Published:** 2013-08-14

**Authors:** Robert Walker, Sarah Derrett, John Campbell, Mark R Marshall, Andrew Henderson, John Schollum, Sheila Williams, Bronwen McNoe

**Affiliations:** 1 Department of Medicine, University of Otago, Dunedin, New Zealand; 2 Department of Preventive and Social Medicine, University of Otago, Dunedin, New Zealand; 3 Renal Medicine, Counties Manukau District Health Board, Manukau, New Zealand; 4 Consultant Nephrologist Renal Unit, Ninewells Hospital, Dundee, Scotland

**Keywords:** Quality of life, Kidney disease, Dialysis, Elderly

## Abstract

**Background:**

The number of elderly people over the age of 65 commencing dialysis in NZ has increased by almost 400% in the past decade. Few data are available about health related outcomes and survival on dialysis in the elderly to help the individual, their family, clinicians and health planners with decision-making.

**Methods/design:**

This study will provide the first comprehensive longitudinal survey of health-related quality of life (HRQOL) and other patient centred outcomes for individuals aged ≥65 years on, or eligible for, dialysis therapy and will link these data to survival outcomes. Data collected by yearly structured interviews with participants will be linked to co-morbidity data, health service use, and laboratory information collected from health records, and analysed with respect to HRQOL and survival. The information obtained will inform the delivery of dialysis services in New Zealand and facilitate improved decision-making by individuals, their family and clinicians, about the appropriateness and impact of dialysis therapy on subsequent health and survival.

**Discussion:**

Results from this study will make possible more informed decision-making by future elderly patients and their families as they contemplate renal replacement therapy. Results will also allow health professionals to more accurately describe the impact of dialysis therapy on quality of life and outcomes for patients.

**Trial registration:**

ACTRN12611000024943.

## Background

There has been an increase of over 400% in the number of elderly and very elderly patients on dialysis in New Zealand over the past 2 decades [1]. This rapid increase has generated considerable debate resulting in wide variation in attitude towards referral and acceptance of elderly patients for dialysis [2-5]. One major reason for this is that there is uncertainty about the outcome from dialysis treatment in this population. Decision-making should, and clearly does, involve the patients and their carers’, along with health service providers. However, there is currently a dearth of evidence related to such decision-making among dialysis patients in general, and elderly dialysis patients in particular [6].

The assumption is often made that dialysis is appropriate for all individuals; however this may not be a valid assumption. Dialysis by the nature of the intervention has a large potential to negatively influence the quality of life of the individual and immediate family. While dialysis usually prolongs life in people with end stage kidney disease (ESKD), it has also been found that both patients and their partners are overwhelmed by the impact of dialysis on their lives [4]. Dialysis may substantially worsen quality of life especially in those with significant co-morbidity. However, if patients opt for conservative therapy (no dialysis) it is unknown how much life expectancy and quality of life is actually altered. There are currently few scientific data to inform and help clinicians, their patients and family to make a decision.

In addition to the impact of dialysis treatment on the individual, there is a significant health-economic implication for NZ, with the approximate cost of dialysis per individual at about $65,000 to $80,000 per year. In New Zealand, this equates to approximately $56 million per year for those on dialysis over the age of 65 years. This cost does not include the cost of hospitalisation and the impact of any other associated co-morbidity that is frequently present in the elderly and may be exacerbated by ESKD and/or its treatment. Although the crude cost for renal replacement therapy (RRT) can currently be estimated, there is no information about patient-experienced benefits to the individuals beyond survival.

The determinants of successful dialysis in the elderly will be multifactorial including the degree of autonomy or control related to managing dialysis (home care versus satellite or in-centre based care), and the many socio-economic factors related to the management of a chronic disease superimposed upon the aging process.

A recent study from a large London dialysis centre looked at outcomes between two groups of older patients, one group that opted for dialysis therapy and the other that chose maximal conservative care [6]. Those opting for conservative care were older (mean age 82 years versus 76 years). Although the dialysis group survived for a longer period (mean 2 years), the majority in the conservative group survived for over 13 months with substantially lower hospital days (16 days/per patient/year) and the majority in this group died at home [6]. The dialysis patients were dialysed in a hospital centre, that meant these patients averaged 173 days/per patient/year at the hospital. This study did not record any QoL assessment, data related to patient satisfaction, cost-effectiveness or the socio-economic impact of the hospital-based treatment [6].

In a thematic analysis of the literature Morton and colleagues demonstrated that awareness of factors associated with decision-making related to the management of CKD can provide health professionals with evidence on how best to deliver education programmes for patients and their family, as well as enhancing the patient and their family’s capacity to share in that decision making process [7]. They identified 4 themes – confronting mortality (life/death, burden on family, state of limbo), lack of choice (options not always discussed), knowledge of options, and weighing the alternatives [7]. These are the important issues that this study will focus upon.

It is vital for future health care delivery of renal replacement therapy in those aged ≥65 years in New Zealand that reliable data are obtained. It is also important to have accurate data upon which to base priority decisions regarding health funding and outcomes. In 2008, there were 154 new patients over 65 years commencing dialysis. This is a rate of 397 per million for elderly patients, compared to the overall rate of new patients for New Zealand at 109 per million [1].

### Aims

1) To determine the impact of age, sex, ethnicity, duration of dialysis, satisfaction with health services and co-morbidity on the health-related quality of life (HRQoL) in elderly patients (≥65 years) with chronic and end-stage kidney disease (CKD, ESKD).

2) Compare and contrast survival, health service utilisation, costs and HRQoL outcomes of older patients with EKSD according to the type of renal replacement therapy including modality (haemodialysis versus peritoneal dialysis) and location (home versus facility) or maximal conservative therapy (no dialysis).

3) To develop evidence-based guidelines for optimal management of older patients with severe CKD and ESKD.

### Hypotheses

From the cross-sectional data at baseline, we hypothesise that HRQoL differs between patients according to: sex, ethnicity, co-morbidities, type of dialysis treatment, health service satisfaction, costs, family support and duration of dialysis.

From longitudinal data in Years 2 and 3, we hypothesise that: HRQoL is positively associated with survival and that the HRQoL trajectory will differ between patients of different sex, ethnicity, co-morbidities, type of dialysis, availability of family and community support and duration of dialysis.

## Methods/Design

### Study design

The study uses an “accelerated longitudinal design”, comprised of cross-sectional and longitudinal components. The cross-sectional data are collected at the baseline interview and from clinical records in Year 1. The longitudinal data are collected from interviews 2 and 3 (12 and 24-months after the initial interview) and from clinical records in Years 2 and 3. This study design is optimal since it: 1) allows for the provision of timely results from an initial cross sectional analysis, and 2) by including the prevalent dialysis patients at baseline and the incident patients eligible for dialysis each year, it also allows for sufficiently powered longitudinal analyses, without the need for extended recruitment that would be necessary if the study used a dialysis inception cohort.

The nephrology teams responsible for their medical care have undertaken the identification of potential participants. All eligible (and consenting) participants will be contacted by telephone to arrange an interview. Data will be collected at each of the three interviews using structured questionnaires administered to participants by trained interviewers and from clinical records by the research nurses. The first Baseline Interview will be administered to all eligible participants already on dialysis for at least 90 days at the start of the study and to all new patients who are eligible for dialysis each year. Follow-up questionnaires are then administered to survivors 12 and 24-months later. The questionnaires have been piloted with 60 older patients with CKD and found to be acceptable and to cover questions and issues of greatest relevance to them [8]. Each interview will be designed to take 45-60 minutes and will be conducted either on the telephone or face to face depending on patient preference.

### Study participants

**Figure 1:**
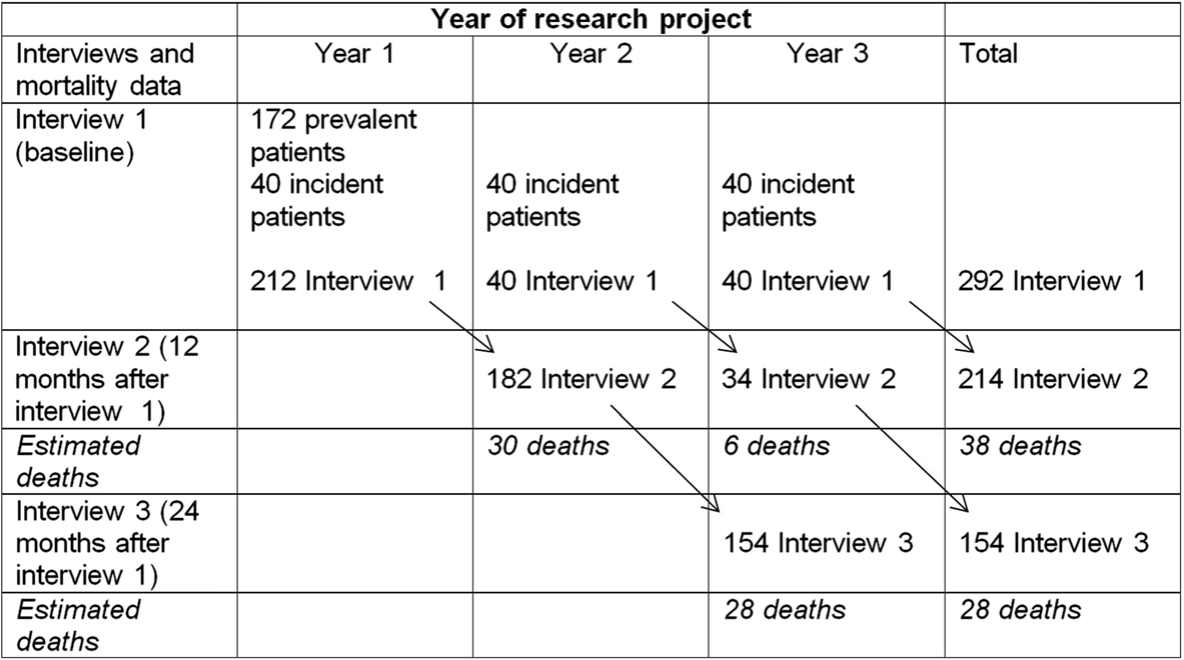
Presenting Recruitment and Follow-up (assuming an 70% response rate).

Our cohort will consist of dialysis and pre-dialysis patients recruited from three services across New Zealand, each serving different patient populations using contrasting models of care for dialysis delivery. These are: Middlemore Hospital (Counties Manukau District Health Board (DHB)), the largest dialysis service in New Zealand, providing both home and facility-based dialysis in mostly urban settings including a large proportion of New Zealand Māori and Pacific population; Dunedin Hospital (Southern DHB), a medium sized dialysis service providing entirely home-based dialysis in mixed urban and rural settings; and Hastings Hospital (Hawkes Bay DHB), a small sized dialysis service providing both home and facility-based dialysis in mostly rural settings. There is always a tension between the number of sites and speed of recruitment and the desire to carefully manage recruitment and the study process. By recruiting from three sites we ensure a range of participant characteristics in the study that are representative of New Zealand, while also ensuring that the study is efficient in terms of project management and data analysis. Using registry data from the Australian & New Zealand Dialysis and Transplant registry (ANZDATA), and a recruitment rate of 70%, it is estimated that we will enroll 160 to 172 prevelant participants and approximately 40 incident participants in the first year (Figure 1).

All patients will be followed prospectively including those who decide not to undertake dialysis, to explore changes in their HRQoL and survival status. The effects of age, sex, ethnicity, co-morbidities, modality, duration of dialysis treatment, family support and prior HRQoL on current quality of life and survival will be examined. Interview questionnaires include the SF-36 [9], the Kidney Disease Questionnaire [10] (KDQoL) EQ-5D [11,12]), WHODAS [13]. Inclusion of the KDQoL will make possible the comparison of the SF-36 health status of participants with other groups within New Zealand and will also provide kidney disease-specific information for comparing outcomes with international studies [14-17].

### Inclusion criteria

1) Prevalent dialysis patients under the three dialysis services aged ≥65 years who have been on dialysis for 90 days prior to survey date will be eligible to participate in the study.

2) Incident chronic kidney disease patients aged ≥65 years with an eGFR ≤15 ml/min/1.73 m^2^ presenting to the same services for consideration of dialysis will be eligible to participate in the study. A proportion of these patients will decide not to undertake dialysis. They will provide important data to compare and contrast with those who commence dialysis.

3) Patients aged ≥65 years who present with hitherto unrecognized renal failure requiring immediate dialysis will be eligible if their renal failure is deemed permanent by the treating nephrologist, and if dialysis is intended to be an indefinite treatment.

### Exclusion criteria

1) Inability to give informed consent

2) Inability to participate in a telephone or face-to-face interview

3) Inter current illness requiring hospitalisation (current and within 2 weeks of the survey period) and of sufficient severity to affect the patient’s ability to take part in the opinion of the treating physician (interviews for this last group will be rescheduled for 1 month later). Note that any selection bias introduced by this exclusion will be explored by comparison of survival of all patients from each unit.

### Data collected

#### Questionnaire

Most questions within the structured questionnaire are closed-response questions amenable to quantitative analysis. Most of these questions were trialled in a pilot study by us and found to be acceptable to patients [8]. The structured questionnaires also collect some information in ‘free-text/open-ended response’ format about participants’ decision-making about commencing dialysis, and dialysis type (haemodialysis or peritoneal) and their experience and perceptions of dialysis which will be analysed thematically. The interviews comprise

1. *Kidney disease* - The shorter version KDQol, [18-20] which includes the SF-36 [9] and Kidney Disease Questionnaire [10]. Inclusion of the KDQoL will make possible the comparison of the SF-36 health status of participants with other groups within New Zealand and also will provide kidney disease-specific information for comparing outcomes with international studies [14-17]. The KDQoL also includes a number of questions specifically enquiring about: managing kidney disease, CKD symptoms, effect of CKD on daily life and satisfaction with dialysis care.

2. *General Health Status* - EQ-5D. It is applicable to a wide range of health conditions and treatments; it provides a simple descriptive profile and rating of health status of use for within-study group comparison, economic analyses and for international comparison of health status outcomes [21]. New Zealand population norms are available for the EQ-5D (25) [22]. The alcohol use disorders identification test (AUDIT) was used to assess alcohol consumption [23] and some additional questions on illicit drug use using the AUDIT were added.

3. *Disability* - The WHODAS II, a short 12-question of measure of disability is one of few measures specifically designed to measure disability according to the World Health Organisation ICF model of disability [24]. Subjective wellbeing was measured using the Australian instrument, the Personal Well-Being Index [25].

4. *Social Supports:* 1) Residential status, including residential care and extended family support; 2) ability to perform independent dialysis treatment and/or any assistance required from family or friends; 3) what, if any, help is required by the patient around the home.

5. *Economic Burden and Personal Costs:* Three questions, modelled on the Ministry of Social Development sponsored study into the Living Standards of Older People [26] to discover financial burden for comparison with data from the general population. Information will also be collected about personal costs incurred as a result of dialysis [8].

6. *Mental health:* Depressive symptoms are common in chronic disease and have been shown to be related to survival in some studies [27] but not in others [28]. The relationship between depression and survival in the elderly dialysis population remains to be defined and there may be a positive relationship between age and emotional state [29]. We are measuring this with questions from the SF-36 scale and a question from the EQ-5D [9].

7. *Demographic data:* Data about ethnicity, descent from New Zealand Māori, household composition and home ownership will be collected as per the New Zealand Census [30].

#### Clinical records

1. *Co-morbidity:*Following participants consent, research staff will collect these data from participants’ clinical records. Co-morbidity may have significant influence on outcome with adverse effects on survival depending on specific conditions like diabetes, ischaemic heart disease and peripheral vascular disease [14]. Several indices of co-morbidity have been used for patients with chronic kidney disease and dialysis populations, for example: the Khan [31], Davies [32], and the Charlson [33] indices. However these were derived for general populations, not specifically CKD populations. We plan to utilise a newer improved co-morbidity index, validated against the USRDS end stage renal failure population, that is simpler, easier to use and has been shown to outperform the Charlson co-morbidity index [34]. It applies numerical weights to co-morbid conditions including atherosclerotic heart disease, congestive heart failure, cardiovascular disease, peripheral vascular disease, dysrhythmias, chronic airways disease, liver disease, cancer and diabetes. The sum of the weights present for the individual can be used as a continuous variable in analyses [34]. This will be linked to health service utilisation and adverse outcomes.

2. *Survival, other adverse outcomes, laboratory data and health service utilisation:* Data about survival, adverse outcomes, laboratory data and health service utilisation (including hospital admissions and length of stay) will also be collected from clinical records.

### Trial registration

Australian and New Zealand clinical trials registry: ACTRN12611000024943.

### Ethical approval

The study protocol was approved by the New Zealand Multi-Regional Ethics Committee, approval number MEC/10/084.

### Data analysis

All analyses will be performed using Stata 11.0 (or subsequent versions). Confidence intervals will be provided in all cases. All data will be described using appropriate summary statistics and frequency tables where necessary. Associations between the variables will be assessed in terms of statistical significance at a level of P <0.05.

### Cross sectional analysis from baseline data

Linear mixed models will be used to model quality of life measures (accounting for clustering within centres) based on subject demographics (including age, sex, ethnicity, socioeconomic status), co-morbidities, and modality and duration of dialysis. The prevalent dialysis participants will be grouped for analysis according to their vintage on dialysis, and combined for analysis with the incident patients in Year 1. Differences between categorical measures (e.g., ethnicity) will be assessed using adjusted means. Relationships between continuous measures (e.g., quality of life and dialysis duration) will be assessed using correlations. The study is powered at 80% to detect differences between means of 0.55 standard deviations for two groups sized 106 patients each (half of the sample), and differences of 0.8 standard deviations between two groups sized 53 patients each (quarter of the sample). These detectable differences are considered to be of clinical importance.

### Longitudinal analysis

Mixed models will be used for the majority of the longitudinal analyses to estimate the trajectory of HRQoL over time. Correlation between repeated measures within centres and participant will be accounted for. Differences between groups at different time points will be assessed using adjusted means. Assuming a design effect of 2 for the quality of life instruments in the target population and a correlation of 0.65 between a participant’s repeated measurements, the study would be powered at 80% to detect changes as small as 0.45 standard deviations per six-month period within a group containing 38 patients.

### Survival analysis

With the high mortality rate, 24 months of follow up of this cohort will have sufficient power to determine impact on survival. It will identify which key HRQoL measures provide the best discrimination and calibration in determining outcome, so that an abbreviated assessment can potentially be incorporated into evidence-based guidelines to clinical practice. The survival analysis will be performed initially using Cox’s proportional hazards regression which has the potential to detect a hazard ratio of 0.8 with 80% power, and p <0.05 will be considered significant.

## Discussion

This project will clearly address the relevance, timeliness, and the sustainability of dialysis in the older age group. The provision of dialysis, preferably in a home setting or low level self-care satellite units closer to the individuals’ residences, may allow better integration with primary and community care. If conservative management is shown to be an important and valid option with similar outcomes to dialysis, then this can be appropriately discussed with the individual and their family without this being considered as rationing, or limiting health resources, to this age group (which is often a perception). Elderly patients who do not commence dialysis often survive many months (6), and some may have a better quality of life (HRQoL).

Providing information as to preferred options by this age group related to their expectations and perceived quality of life will immediately influence delivery of healthcare. The data generated by this study will critically inform health care for the older age group.

Results from this study will make possible more informed decision making by future elderly patients and their families as they contemplate renal replacement therapy. Results will also allow health professionals to more accurately describe the impact of dialysis therapy on quality of life and outcomes for patients.

Funding was received from the New Zealand Health Research Council in 2010 (HRC 10/354; NZD$775,000). A publication reporting early data from the first year of the project is currently being prepared for publication.

## Competing interests

All authors declared that they have no competing interests.

## Authors’ contribution

RW is the Principal Investigator. RW, SD and BM drafted the manuscript. All authors contributed to the study design and were named investigators on the grant. All authors read and approved the final manuscript.

## Pre-publication history

The pre-publication history for this paper can be accessed here:

http://www.biomedcentral.com/1471-2369/14/175/prepub
